# A 12-Week Prospective, Double-Blind, Multicenter, Randomized Study Comparing 100 Units of Abobotulinum Toxin Type A (Dysport^®^) and 33.33 Units of Neubotulinum Toxin Type A (Neuronox^®^) for the Treatment of Hemifacial Spasm

**DOI:** 10.3390/toxins17040173

**Published:** 2025-04-02

**Authors:** Subsai Kongsaengdao, Arkhom Arayawichanont, Kanoksri Samintharapanya, Pichai Rojanapitayakorn, Benchalak Maneeton, Narong Maneeton

**Affiliations:** 1 Office of Senior Advisor, Department of Medical Services, Ministry of Public Health, Nonthaburi 11000, Thailand; 2 Division of Neurology, Rajavithi Hospital, Department of Medical Services, Ministry of Public Health, Bangkok 10400, Thailand; 3 Division of Neurology, Department of Medicine, College of Medicine, Rangsit University, Bangkok 10400, Thailand; 4 Division of Neurology, Department of Medicine, Sunpasitthiprasong Hospital, Ubon Ratchathani 34000, Thailand; aarayawi@gmail.com; 5 Division of Neurology, Department of Medicine Lampang Hospital, Lampang 52000, Thailand; tem2000_1@yahoo.com; 6 Division of Neurology, Department of Medicine Suratthani Hospital, Suratthani 84000, Thailand; pichairoj2518@gmail.com; 7 Department of Psychiatry, Faculty of Medicine, Chiang Mai University, Chiang Mai 50200, Thailand; benchalak.maneeton@cmu.ac.th (B.M.); narong.m@cmu.ac.th (N.M.)

**Keywords:** hemifacial spasm, botulinum Toxin A, quality of life, sleep quality, depression

## Abstract

Previous randomized controlled trials (RCTs) investigating Botulinum toxin A (BoNT-A) for treatment of hemifacial spasm (HFS) have primarily focused on symptom relief and quality-of-life improvement. However, head-to-head comparisons of different BoNT-A formulations, particularly in terms of onset, duration of action, and efficacy, remain limited. We conducted a 12-week prospective, randomized controlled trial comparing the efficacy and safety of 33.33 units of Neubotulinum toxin A (Neu-BoNT-A) with 100 units of Abobotulinum toxin A (Abo-BoNT-A) in the treatment of HFS. A total of 87 patients were enrolled between September and December 2024. Neu-BoNT-A and Abo-BoNT-A exhibited similar onset and duration of action [5.0 ± 0.9 vs. 6.2 ± 0.7 days, respectively (*p* = 0.33)]. After 12 weeks of treatment, Neu-BoNT-A demonstrated superior efficacy in reducing the daily duration of HFS (2.00 ± 0.06 vs. 1.42 ± 0.10 h/day, *p* < 0.001) and improving sleep duration (1.37 ± 0.01 vs. 1.06 ± 0.01 h/day, *p* < 0.001). However, Abo-BoNT-A was associated with significantly lower absolute daily disability time compared to Neu-BoNT-A (11.4 vs. 1.2 min/day, *p* < 0.001). No serious adverse events were observed. Both Neu-BoNT-A and Abo-BoNT-A were safe and effective in treating HFS. However, Neu-BoNT-A was more effective in HFS with minimal symptoms without disability and Abo-BoNT-A more effective in HFS with greater duration of disability.

## 1. Introduction

Previous randomized controlled trials (RCTs) investigating the use of Botulinum toxin A (BoNT-A) for treating hemifacial spasm (HFS) have primarily focused on evaluating its efficacy in relieving symptoms, reducing the pain and discomfort caused by twitching, and improving patients’ quality of life [1,2,3,4]. To date, only four prospective, double-blind RCTs have reported the duration of action of BoNT-A in HFS treatment. Lolekha (2017) [1] reported that Ona-botulinum toxin A (Ona-BoNT-A/Botox^®^) had a duration of action of 72 days, while Rieder (2007) [2] and Quagliato (2010) [3] found durations of 71 days and 89.6 days, respectively. Kongsaengdao (2012) [4] demonstrated that Neu-BoNT-A and Abo-BoNT-A exhibited a duration of action of 84 days using patient symptom diaries.

Few studies have compared the dosages of BoNT-A in HFS treatment [3,4], while the dosage ratio of Abo-BoNT-A to Ona-BoNT-A has been approximated at 1:3 [4,5]. Only one RCT has compared Neu-BoNT-A and Abo-BoNT-A using an approximate ratio of 1:5 [4]. All published studies report that BoNT-A, including Ona-BoNT-A, Abo-BoNT-A, and Neu-BoNT-A, is highly effective in reducing facial twitching and significantly improving patients’ quality of life [1,2,3,4].

To date, no studies have directly compared the clinical efficacy and quality of life outcomes using an equivalent dose ratio (1:3) of 100 units of Abo-BoNT-A and 33.33 units of Neu-BoNT-A in patients with HFS. This study is the first to investigate the efficacy and safety of a 1:3 dose ratio of Neu-BoNT-A to Abo-BoNT-A, compared to the previously studied 1:5 dose ratio, in which Neu-BoNT-A demonstrated inferiority to Abo-BoNT-A. Utilizing a 12-week, prospective, double-blind, randomized controlled trial design, we aimed to evaluate whether the adjusted dose ratio improved clinical outcomes. We hypothesized that increasing the dose of Neu-BoNT-A may enhance clinical efficacy compared to the previously established 1:5 dose ratio of Abo-BoNT-A. This study directly compares the efficacy and safety of Abo-BoNT-A at a total dose of 100 units and Neu-BoNT-A at a total dose of 33.33 units in reducing the daily duration of facial muscle spasms, disability duration, functional impairment, and associated safety outcomes in patients with HFS over a 12-week treatment period.

## 2. Results

### 2.1. Demographics and Baseline Characteristics

A total of 87 primary hemifacial spasm (HFS) patients (67 females, 20 males) were enrolled from September to December 2024. All patients completed the study. The mean (SD) patient age was 61.24 (10.4) years (range: 35–80 years), with a mean age of first diagnosis of 54.0 (8.0) years (range: 26–77 years). The mean (SD) duration of hemifacial spasm was 7.4 (4.3) years (range: 1 month–13 years), and the mean (SD) duration of previous HFS treatment with BoNT-A was 7.4 (4.3) years (range: 0–13 years). Among participants, 85 had previously been treated with Abo-BoNT-A, 26 had a previous history of treatment with both Abo-BoNT-A and Neu-BoNT-A, and two were BoNT-A naïve. No patients had significant medical comorbidities. Totals of 42 and 45 patients were randomized to receive treatment with Neu-BoNT-A 33.33 units and Abo-BoNT-A 100 units, respectively.

### 2.2. Primary Outcomes (Table 1)

**Figure 1:**
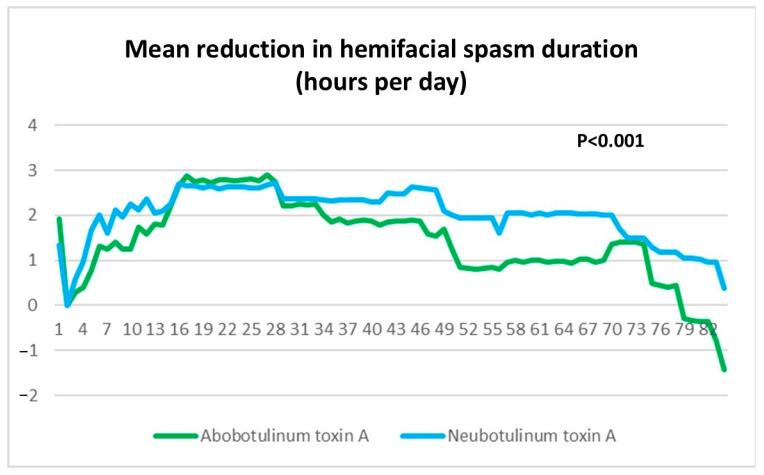
Reduction in hemifacial spasm duration (hours/day) from baseline following treatment with Neu-BoNT-A 33.33 units (blue line) compared to Abo-BoNT-A 100 units (green line). The X-axis represents the days after treatment, and the Y-axis indicates the number of hours per day of hemifacial spasm duration reduced from baseline. Neu-BoNT-A demonstrated significantly greater efficacy in reducing hemifacial spasm duration compared to Abo-BoNT-A (*p* < 0.001).

Reduction in Hemifacial Spasm Duration: Neu-BoNT-A demonstrated superior efficacy in reducing hemifacial spasm duration from baseline compared to Abo-BoNT-A at all time points [0–4 weeks: 2.13 ± 0.13 vs. 1.97 ± 0.16 h/day (*p* = 0.04), 4–8 weeks: 2.28 ± 0.04 vs. 1.66 ± 0.09 h/day (*p* < 0.001), 8–12 weeks: 1.60 ± 0.09 vs. 0.63 ± 0.13 h/day (*p* < 0.001), and overall (0–12 weeks): 2.00 ± 0.06 vs. 1.42 ± 0.10 h/day (*p* < 0.001)]. Neu-BoNT-A demonstrated significantly greater efficacy in reducing hemifacial spasm duration compared to Abo-BoNT-A at all assessed 24 h time points after treatment (Figure 1). Additionally, Neu-BoNT-A (33.33 units) demonstrated superiority over Abo-BoNT-A (100 units) in absolute mean hemifacial spasm duration per day during weeks 0–4 (2.83 ± 0.13 h/day vs. 4.73 ± 0.16 h/day; *p* < 0.001), weeks 4–8 (2.68 ± 0.04 h/day vs. 5.03 ± 0.09 h/day), weeks 8–12 (3.36 ± 0.09 h/day vs. 6.06 ± 0.13 h/day; *p* < 0.001), and overall (0–12 weeks): 2.96 ± 0.06 vs. 5.27 ± 0.10 h/day (*p* < 0.001).

**Figure 2:**
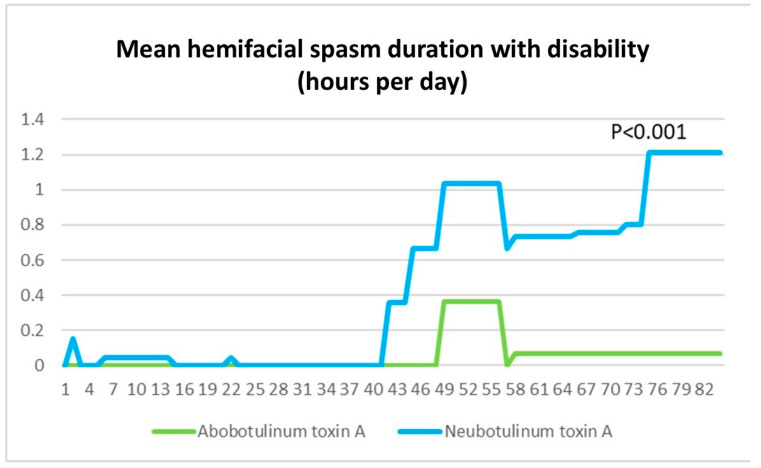
The absolute disability duration in hemifacial spasm (hours/day) following treatment with Neu-BoNT-A 33.33 units (blue line) compared to Abo-BoNT-A 100 units (green line). The X-axis represents the days after treatment, and the Y-axis indicates the number of hours per day of hemifacial spasm duration reduced from baseline. Abo-BoNT-A demonstrated significantly greater efficacy in reducing hemifacial spasm duration with disability compared to Neu-BoNT-A at all assessed 24 h time points after treatment.

However, in HFS patients with disability, Neu-BoNT-A showed a smaller reduction in absolute disability duration compared to Abo-BoNT-A [0–4 weeks: 0.01 ± 0.00 vs. 0.00 ± 0.00 h/day (36 vs. 0 s; *p* < 0.001), 4–8 weeks: 0.16 ± 0.03 vs. 0.05 ± 0.01 h/day (9.6 vs. 3 min; *p* < 0.001), 8–12 weeks: 0.42 ± 0.02 vs. 0.03 ± 0.00 h/day (25.2 vs. 1.8 min; *p* < 0.001), and overall (0–12 weeks): 0.19 ± 0.02 vs. 0.02 ± 0.00 h/day (11.4 vs. 1.2 min; *p* < 0.001)]. Abo-BoNT-A demonstrated significantly greater efficacy in reducing hemifacial spasm duration with disability compared to Neu-BoNT-A at all assessed 24 h time points after treatment (*p* < 0.001) (Figure 2).

**Figure 3:**
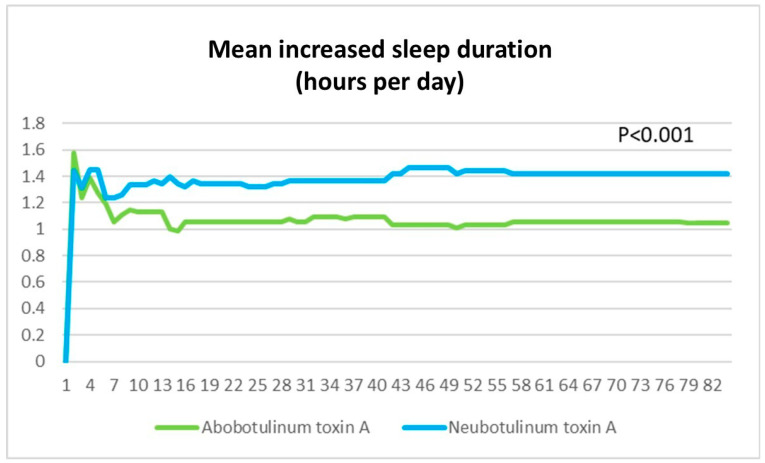
Increase in sleep duration (hours/day) from baseline in hemifacial spasm patients following treatment with Neu-BoNT-A 33.33 units (blue line) compared to Abo-BoNT-A 100 units (green line). The X-axis represents the days after treatment, and the Y-axis shows the increase in sleep duration (hours/day) relative to baseline. Neu-BoNT-A demonstrated a smaller reduction in absolute disability duration compared to Abo-BoNT-A at all time points (*p* < 0.001).

Sleep Duration: Improved sleep duration was significantly greater with Neu-BoNT-A [0–4 weeks: 1.29 ± 0.04 vs. 1.07 ± 0.04 h/day (*p* < 0.001), 4–8 weeks: 1.41 ± 0.00 vs. 1.05 ± 0.00 h/day (*p* < 0.001), 8–12 weeks: 1.41 ± 0.00 vs. 1.05 ± 0.00 h/day (*p* < 0.001), and overall (0–12 weeks): 1.37 ± 0.01 vs. 1.06 ± 0.01 h/day (*p* < 0.001)]. Neu-BoNT-A demonstrated significantly greater efficacy in absolute sleep duration compared to Abo-BoNT-A at all assessed 24 h time points after treatment (Figure 3).

Onset and Duration of Action: There were no significant differences in onset or duration of action between Neu-BoNT-A and Abo-BoNT-A [Onset of action: 5.0 ± 0.9 vs. 6.2 ± 0.7 days (*p* = 0.33), and Duration of action: 74.7 ± 2.4 vs. 75.7 ± 1.8 days (*p* = 0.71)].

### 2.3. Secondary Outcomes

Quality of Life and Depression: No significant differences were observed between Neu-BoNT-A and Abo-BoNT-A in HFS-30, AIMS, or CES-D scores (*p* > 0.05). There was no evidence of depression (CES-D cutoff ≥ 20) or differences in PHQ-9 scores between the groups (*p* > 0.05).

Adverse Events: Adverse events were rare and similar between groups, including unilateral eyelid ptosis (n = 2), unilateral lip ptosis (n = 1), unilateral drooling (n = 1), and acute migraine (n = 1). There was no difference in adverse events between the groups (*p* > 0.05).

## 3. Discussion

This study was designed as a non-inferiority trial to compare the efficacy and duration of action of Neu-BoNT-A and Abo-BoNT-A at a 1:3 dose ratio [4]. Our findings support previous evidence demonstrating the comparability of these two formulations while also providing novel insights into their onset and duration of action. Notably, Abo-BoNT-A exhibited greater efficacy within the first 12 weeks post-treatment in patients with severe functional disability, suggesting a more pronounced early therapeutic effect in this subgroup. In contrast, Neu-BoNT-A appeared to have a prolonged duration of action in patients without significant disability. These findings indicate potential differences in the biological properties of these formulations, highlighting the need for further investigation into their molecular mechanisms and clinical implications in future studies.

Previous studies have demonstrated the efficacy of Abo-BoNT-A (100 units) over Neu-BoNT-A (20 units) in absolute mean hemifacial spasm duration over a 4-week period (3.64 ± 0.4 h/day vs. 4.7 ± 0.4 h/day) [4]. In contrast, the present study showed that Neu-BoNT-A (33.33 units) was superior to Abo-BoNT-A (100 units) in absolute mean hemifacial spasm duration per day over 4 weeks (2.83 ± 0.13 h/day vs. 4.73 ± 0.16 h/day; *p* < 0.001). Both studies employed a 24 h diary to record the duration of hemifacial spasms and to evaluate the dose–response relationship of Neu-BoNT-A when compared to Abo-BoNT-A. Additionally, Neu-BoNT-A (33.33 units) demonstrated superiority over Abo-BoNT-A (100 units) in reducing mean hemifacial spasm duration per day during weeks 0–4, weeks 4–8, and weeks 8–12 (*p* < 0.001). These findings indicate that Neu-BoNT-A (33.33 units) possesses greater efficacy than Abo-BoNT-A (100 units) in reducing hemifacial spasm duration over a 12-week treatment period. Consistent with conversion ratios previously established, 3 units of Abo-BoNT-A (Dysport^®^) are equivalent to 1 unit of Ona-BoNT-A (Botox^®^), suggesting that 100 units of Abo-BoNT-A should be compared with 33.33 units of Neu-BoNT-A (Neuronox^®^) for comparable efficacy in treatment of HFS.

Regarding the duration of action, earlier studies have reported that Ona-BoNT-A (Botox^®^) has a mean duration of efficacy in hemifacial spasm of approximately 64.3 days, compared to 41.8 days for Abo-BoNT-A (Dysport^®^). Subsequent investigations, including those by Lolekha et al. (2017) [1], Rieder et al. (2007) [2], and Quagliato et al. (2010) [3], revealed durations of action for Ona-BoNT-A (Botox^®^) ranging from 71 to 89.6 days. Similarly, Kongsaengdao et al. (2012) [4] demonstrated that Neu-BoNT-A (Neuronox^®^) at a dose of 20 units exhibited a duration of action comparable to Ona-BoNT-A (Botox^®^), with a median duration of 84 days.

Regarding the potency of Neu-BoNT-A compared to Abo-BoNT-A in this study, patients with a longer duration of hemifacial spasm without disability demonstrated a better response to Neu-BoNT-A. Conversely, in patients with more severe HFS with disability, Abo-BoNT-A showed superior efficacy in reducing the duration of disability. Baseline severity data were incorporated into the analysis, and although the groups were not perfectly matched, the Abo-BoNT-A group exhibited greater baseline disability, with a mean spasm duration of 4.78 ± 0.63 h per day compared to 3.63 ± 0.62 h per day in the Neu-BoNT-A group (*p* = 0.001). Despite this initial imbalance, the final results demonstrated a greater reduction in disability in the Abo-BoNT-A group, supporting its superior efficacy relative to Neu-BoNT-A. This potential source of bias will be acknowledged as a study limitation.

The findings of this study align with previous research that employed similar methodologies, study designs, and measurement techniques in a comparable population of chronic hemifacial spasm (HFS) patients with a history of prior botulinum toxin treatment. The patient characteristics in both studies were consistent, supporting the generalizability of our results. The superiority of Neu-BoNT/A at a 1:3 dilution ratio was demonstrated through its concentration-dependent effect, which was not observed at a 1:5 dilution. This suggests that dilution ratios play a critical role in determining the efficacy and diffusion properties of botulinum toxin formulations. Different commercial botulinum toxin formulations, such as Abo-BoNT-A and Neu-BoNT-A, exhibit distinct diffusion profiles due to variations in protein complex size and excipients. Abo-BoNT-A’s molecular profile, as assessed by size-exclusion high-performance liquid chromatography (SE-HPLC), reveals a primary peak corresponding to the 150 kDa botulinum neurotoxin, along with additional peaks representing associated non-toxic accessory proteins. These structural characteristics contribute to its greater diffusion capacity compared to Neu-BoNT-A. In contrast, Neu-BoNT-A produces a single peak in SE-HPLC analysis, with an average molecular weight of 904 kDa, indicating a larger molecular complex that may limit its diffusion. The diffusion properties of these formulations are particularly relevant in patients with more severe disability, where a higher diffusion capacity may enhance clinical efficacy. Given Neu-BoNT-A has a larger molecular structure and reduced diffusion, a higher concentration may be required to achieve comparable effects, especially in cases where broader toxin spread is beneficial. These findings highlight the importance of selecting an optimal formulation and dilution ratio based on the desired therapeutic outcome.

To further investigate these findings, future studies should evaluate the potency of Neu-BoNT-A relative to Abo-BoNT-A using standardized assays, including Light-Change Activity High-Performance Liquid Chromatography (LCA-HPLC), cell-based potency assays, compound muscle action potential (CMAP), and digital abduction score (DAS) assays under identical experimental conditions. Clinically, these results suggest that Abo-BoNT-A may be preferable for patients with greater disability, while Neu-BoNT-A may be more beneficial for those with longer durations of spasms.

The current study found no significant differences in the onset of action (5.0 ± 0.9 vs. 6.2 ± 0.7 days; *p* = 0.33) or duration of action (74.7 ± 2.4 vs. 75.7 ± 1.8 days; *p* = 0.71) between Neu-BoNT-A (33.33 units) and Abo-BoNT-A (100 units). Notably, this study is one of the first randomized controlled trials to compare these two dosages of botulinum toxin A formulations in a head-to-head manner.

To minimize confounding factors, major interfering medications known to affect hemifacial spasm (HFS) severity were excluded from this study. Additionally, lifestyle factors such as stress, which could influence symptom severity, were assessed and found to be comparable between groups. The SF-36 questionnaire was utilized to evaluate both physical and emotional well-being, confirming no significant baseline differences between the groups. Specifically, domains such as Physical Functioning (PF), Role Physical (RP), Bodily Pain (BP), and General Health (GH) ensured that physical health status was similar, while Vitality (VT), Social Functioning (SF), Role Emotional (RE), and Mental Health (MH) confirmed comparable emotional and psychological well-being. Furthermore, dietary intake, baseline physical status, and sleep duration were evaluated through detailed history-taking, with no significant differences observed between groups. Notably, no patients with a history of botulinum toxin resistance were identified in this study. These factors enhance the validity of our findings by ensuring that the treatment effects observed were not confounded by baseline lifestyle differences or preexisting resistance to botulinum toxin.

There were no differences in health-related quality of life (HRQoL) between Abo-BoNT-A (100 units) and Neu-BoNT-A (20 units) in previous studies. Similarly, the present study demonstrated no significant differences in disease-specific HRQoL outcomes, such as the HFS-30, AIMS, and CES-D scores, between Abo-BoNT-A (100 units) and Neu-BoNT-A (33.33 units).

Interestingly, our study found a female-to-male ratio of 0.77 (67 females vs. 20 males), indicating a female predominance. This finding is consistent with the latest national database on hemifacial spasm (2023), which also demonstrates a higher prevalence in females (7109 females vs. 2108 males; ratio of 0.77). This distribution reflects the real gender ratio of hemifacial spasm in Thailand.

In this study, to ensure consistency and minimize variability within a controlled clinical setting, we adhered to the national protocol, administering a standardized dose to the same muscles, evenly distributed across four injection sites. This approach was primarily designed to enhance safety by limiting the maximum dose per injection site and reducing the risk of adverse effects. However, in routine clinical practice, treatment is often individualized based on the severity of muscle contraction. For the injection sites, we used the same technique at the lateral canthal rhytids, the lateral orbital rim at the level of the lateral canthus with intradermal injection points for the orbicularis oculi muscle, within a layer extending to the superficial musculoaponeurotic system and temporoparietal fascia, at sites positioned approximately 20 mm apart in three directions: superiorly at a 30-degree angle, laterally, and inferiorly at a 30-degree angle to minimize the risk of venous injury, particularly to the sentinel and inferior palpebral veins, which are near the orbital rim. Avoiding injections too close to the orbital rim reduces the risk of serious complications such as diplopia and functional impairment, allowing for adjustments in dosing and injection site selection to optimize therapeutic outcomes [6].

Adverse events associated with botulinum toxin A treatment, such as eyelid ptosis and facial muscle paralysis, may result from off-target effects, particularly in fixed-dose study designs; however, precise anatomical targeting of injection sites can mitigate these risks. Consistent with previously reported safety profiles, this study observed rare adverse events, including unilateral eyelid ptosis, unilateral lip ptosis, unilateral drooling, and acute migraine attacks, with comparable incidence between the two treatment groups.

A potential confounding factor in this study is the residual effect of prior botulinum toxin injections. However, a previous randomized controlled trial comparing Abo-BoNT-A and Neu-BoNT-A at a 1:5 dose ratio reported a duration of response of less than 84 days. In our study, all participants received their last injection at least 14 weeks (98 days) before enrolment, minimizing the likelihood of a significant residual effect. Nonetheless, we acknowledge this as a study limitation, as the majority of participants had chronic hemifacial spasm (HFS) rather than being treatment-naïve. This characteristic of the cohort may introduce bias, which should be considered when interpreting the results.

In this study, all patients had chronic hemifacial spasm (HFS) and underwent neuroimaging with computed tomography (CT) as part of the diagnostic evaluation. While HFS is most commonly attributed to neurovascular compression, the term “primary HFS” was used to distinguish cases without an identifiable secondary cause. We recognize that magnetic resonance imaging (MRI) is the preferred modality for detecting neurovascular compression, and the use of CT instead represents a limitation of this study.

## 4. Conclusions

In summary, Neu-BoNT-A (33.33 units) and Abo-BoNT-A (100 units) demonstrated comparable onset and duration of action, and were effective and safe in patients with hemifacial spasm with minimal adverse events observed. However, Neu-BoNT-A (33.33 units) was superior in reducing hemifacial spasm duration per day and absolute hemifacial spasm duration, and improving sleep duration after 4, 8, and 12 weeks of treatment. In contrast, Abo-BoNT-A (100 units) was associated with a slight advantage in absolute hemifacial spasm duration for patients with more disability. Both formulations were effective and safe for the treatment of hemifacial spasm, with minimal adverse events observed. Future research should include long-term studies exploring the efficacy and safety and alternative dose ratios.

## 5. Materials and Methods

This study aimed to compare the effectiveness of botulinum toxin in terms of the mean reduction of facial spasm duration (hours/day), reduction of disability duration (hours/day), onset of action, duration of action, short-term quality of life, and depression scale scores after 4, 8, and 12 weeks of treatment. The study evaluated 100 units of Abo-BoNT-A versus 33.33 units of Neu-BoNT-A. This phase III, multicenter, 12-week, prospective, double-blinded, randomized controlled trial was conducted from September 2024 to December 2024. The study adhered to the Declaration of Helsinki and International Conference on Harmonization/Good Clinical Practice Guidelines and was approved by the Ethics Committee of Ministry of Public Health of Thailand. Written informed consent was obtained from all participants, and the trial was registered at www.clinicaltrials.gov (accessed on 1 July 2024) (NCT04589364).

### 5.1. Inclusion Criteria

Eligible participants included patients aged 18 to 80, with or without prior exposure to botulinum toxin, who met the diagnostic criteria for primary hemifacial spasm as confirmed by a neurologist. Participants provided informed consent, were conscious, capable of effective communication, and fluent in the Thai language. Female participants of reproductive age were required to avoid pregnancy during the study period and provide a negative urine pregnancy test before enrollment.

All participants were evaluated for their ability to reliably participate in the study, maintain a 24 h treatment diary, and report any side effects or medications used. Eligible participants were in good physical health, able to undergo neurological examinations, and willing to complete standardized questionnaires, including the Abnormal Involuntary Movement Scale (AIMS), the HFS-30 questionnaire, the 36-item Short Form Health Survey (SF-36), the Patient Health Questionnaire-9 (PHQ-9), and the Center for Epidemiologic Studies Depression Scale (CES-D). This comprehensive approach ensured the collection of reliable data to assess the efficacy and safety of the botulinum toxin treatments.

### 5.2. Exclusion Criteria

Patients were excluded from the study if they were pregnant, breastfeeding, or at risk of pregnancy without adequate contraception. Additional exclusions included contraindications or precautions for Botulinum toxin A (BoNT-A) injection, the need for concomitant medications that could interact with BoNT-A (e.g., aminoglycosides, spectinomycin, polymyxin, tetracyclines, lincomycin, or tubocurarine muscle relaxants), a history of allergic reactions to BoNT-A or its components, and inability or unwillingness to fully comply with study requirements, including completing questionnaires. Patients were also excluded if they had received unregistered or experimental drugs within the past six months or had undergone BoNT-A treatment less than 14 weeks (98 days) before the first injection. Additional exclusion criteria included a history of botulism, neuromuscular junction disorders (e.g., myasthenia gravis or Lambert–Eaton syndrome), physical or neurological diseases, or psychiatric disorders that could affect treatment outcomes. These conditions included blood clotting disorders (INR > 1.2), thrombocytopenia, rheumatoid arthritis, coronary artery disease, dementia, psychosis, or any other risk factors for adverse reactions. Patients with a history of substance abuse, planned surgeries during the study period, or those receiving aminoglycosides or other interfering medications were also excluded. Patients meeting the eligibility criteria at the first visit were thoroughly informed about the study, and their medical history and recent/current medications were reviewed.

### 5.3. Study Medication

**Figure 4:**
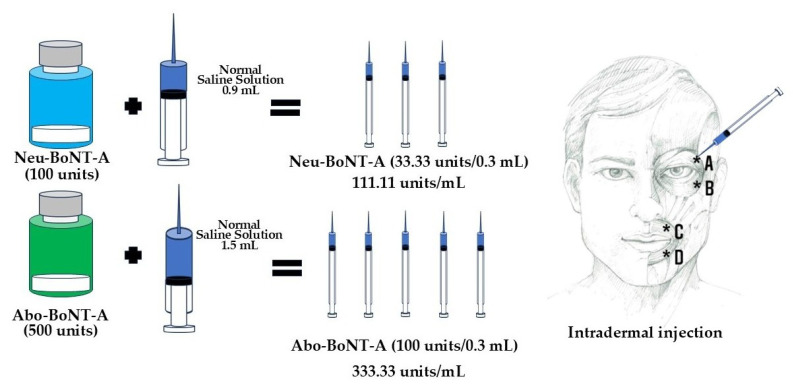
Preparation of Neu-botulinum toxin A (Neu-BoNT-A) and Abo-botulinum toxin A (Abo-BoNT-A), and the intradermal injection sites (*): upper outer border of orbicularis occuli (A); lower outer border of orbicularis oculi (B); upper outer border of orbicularis oris (C); and lower outer border of orbicularis oris (D). Totals of 25 units (0.075 mL) of Abo-BoNT-A or 8.33 units (0.075 mL) of Neu-BoNT-A were injected per injected site.

This study compared low doses of two BoNT-A formulations: Neu-BoNT-A (Neuronox^®^) at 33.33 units and Abo-BoNT-A (Dysport^®^) at 100 units, using a conversion ratio of 1:3 (Neu-BoNT-A 33.33 units: Abo-BoNT-A 100 units) [7,8,9,10]. Both formulations were supplied as freeze-dried powders, reconstituted with normal saline, and used within 2–8 h of preparation (both Neu-BoNT-A and Abo-BoNT-A were refrigerated before and after preparation). Neu-BoNT-A (100 units) was diluted with 0.9 mL of normal saline, yielding approximately 111.11 units/mL, while Abo-BoNT-A (500 units) was diluted with 1.5 mL of normal saline, resulting in 333.33 units/mL. Treatment allocation for Abo-BoNT-A and Neu-BoNT-A was randomized using a computer-generated sequence from https://www.randomizer.org (accessed on 1 July 2024). The randomization codes were printed, sealed in envelopes, and disclosed only to the pharmacist responsible for preparing the botulinum toxin dilutions (Figure 4). After dilution, each treatment was independently double-checked by unblinded staff and assigned a label number specific to each patient to ensure accurate administration. Eligible patients were randomly assigned to receive either Neu-BoNT-A (33.33 units/0.3 mL) or Abo-BoNT-A (100 units/0.3 mL) (Figure 4). Neurologists assessed the symptoms of hemifacial spasm to determine the injection sites. Each patient received intradermal injections (0.075 mL per site) by the same experienced neurologists at four sites: the upper and lower outer borders of the orbicularis oculi and orbicularis oris muscles. Injections were administered during the first visit, with follow-up assessments scheduled throughout the study.

In this study, reconstituted BoNT-A was stored at a controlled temperature of 20–25 °C prior to injection to ensure stability and preserve efficacy. Considering the high ambient temperatures in Thailand, this approach was implemented to minimize the risk of heat-induced degradation. We used a digital thermometer following dilution and throughout the transfer from the pharmacy preparation unit to the clinic at each study site. Notably, the toxin was not subjected to refreezing at any stage, and all diluted BoNT-A was utilized within the recommended timeframe and temperature range, adhering to established guidelines for optimal potency.

### 5.4. Assessments

To ensure objectivity, all patients, assessors, and investigators were blinded during the evaluation process. Participants kept a 24 h hemifacial spasm (HFS) diary and completed standardized questionnaires, including the HFS-30, SF-36, PHQ-9, and CES-D, which were thoroughly explained to them.

#### 5.4.1. Primary Outcomes

The primary outcomes included the mean reduction in HFS duration (hours/day), sleep hours per day, and disability hours per day, measured by using patient-recorded 24 h diaries verified daily by telephone call by the study team. All patients evaluated and documented the onset and duration of action following treatment using a dedicated set of questions in the diary (Appendix A).

#### 5.4.2. Secondary Outcomes

The secondary outcomes included onset and duration of action, health-related quality of life (HRQoL), and depression scores.

##### Disease-Specific HRQoL: HFS-30 Questionnaire [4,11,12]

The HFS-30, a validated and reliable questionnaire, was used to assess disease-specific HRQoL. The Thai version includes 30 items grouped into seven subscales: mobility (5 items), activities of daily living (5 items), communication (3 items), emotional well-being (7 items), stigma (4 items), social support (3 items), and cognition (3 items). Physical health encompasses mobility, daily activities, and communication, while mental health includes emotional well-being, stigma, social support, and cognition.

##### Abnormal Involuntary Movement Scale (AIMS) [4,11]

The AIMS (Thai version) was used to evaluate the severity of involuntary movements. It includes ratings for seven body regions on a 5-point scale, as well as an overall severity score based on amplitude, posture, and movement incapacitation. The scale also considers dental health. Reliability testing in Thai patients at Rajvithi Hospital demonstrated a Cronbach’s alpha > 0.7.

##### General HRQoL Measures

HRQoL was assessed using the Short Form-36 Health Survey (SF-36), which includes eight domains: physical functioning, role limitations due to physical health, role limitations due to emotional problems, vitality, mental health, social functioning, bodily pain, and general health. Scores range from 0 (maximum disability) to 100 (no disability). The Thai version of the SF-36, validated and reliable for Thai patients, was employed [4,11,13].

##### Depression Measures

The Center for Epidemiologic Studies Depression Scale (CES-D) questionnaire was used to evaluate depressive symptoms. It comprises 20 items focusing on various depression-related symptoms (e.g., mood, feelings of guilt, appetite loss, sleep difficulties). Responses range from 0 (rarely) to 3 (most of the time), with total scores ranging from 0 to 60. Scores of 20 or higher indicate a risk of clinical depression, with sensitivity and specificity of 79% and 80%, respectively. The validated Thai version of the CES-D was used [4,11].

The Patient Health Questionnaire-9 (PHQ-9) assessed depressive disorders based on DSM-IV-TR criteria. Each of the nine items is scored from 0 (not at all) to 3 (nearly every day), with a total score ranging from 0 to 27. Severity levels are categorized as minimal (5–9), minor depression/dysthymia/mild major depression (10–14), moderate major depression (15–19), and severe major depression (20+). The validated Thai version was applied in this study [4,11].

This comprehensive evaluation ensured accurate measurement of clinical outcomes, HRQoL, and depressive symptoms in patients with hemifacial spasm.

### 5.5. Statistical Analysis

Demographic data were summarized using mean (standard deviation, SD, and standard error, SE) and range. Outcome variables, including the mean reduction in hemifacial spasm duration (hours/day), disability duration (hours/day), sleep duration, and scores from the HFS-30, AIMS, SF-36, CES-D, and PHQ-9 questionnaires, were presented as mean scores (standard error, SE) at baseline and after 4, 8, and 12 weeks of treatment.

Sample size was calculated using a non-inferiority statistical approach based on the mean reduction in hemifacial spasm duration per day using a non-inferiority design based on clinical relevance and prior studies. The sample size calculation was based on a power of 80% to detect non-inferiority, resulting in 40 participants per group. Changes from baseline in both treatment groups were compared using paired *t*-tests, with a significance level of α = 0.05 and a beta error of 0.8. Data analysis was performed using StatXact version 6.2 Cytel^®^ Studio^®^ (license no. 2060107). Patient randomization was conducted using online software for sealed envelope randomization. The study was registered at ClinicalTrials.gov (identifier: NCT04589364).

## Figures and Tables

**Table 1:** Primary outcome in detail.

Reduced Mean Hemifacial Spasm Duration from Baseline	0–4 Weeks	4–8 Weeks	8–12 Weeks	0–12 Weeks
**Neu-Botulinum toxin A (hours per day)**				
Mean ± Standard Deviation	2.13 ± 0.13	2.28 ± 0.04	1.60 ± 0.09	2.00 ± 0.06
Median [Interquartile Range: IQR]	2.31 [0.65]	2.35 [0.37]	1.84 [0.86]	2.04 [0.55]
Quartile 1–Quartile 3	1.98–2.63	2.05–2.42	1.17–2.03	1.81–2.37
**Abo-Botulinum toxin A (hours per day)**				
Mean ± Standard Deviation	1.97 ± 0.16	1.66 ± 0.09	0.63 ± 0.13	1.42 ± 0.10
Median [Interquartile Range: IQR]	2.06 [1.48]	1.86 [0.51]	0.95 [0.58]	1.40 [0.96]
Quartile 1–Quartile 3	1.28–2.77	1.39–1.90	0.42–1.01	0.94–1.91
***p*** **value**	**0.04**	**<0.001**	**<0.001**	**<0.001**
**Absolute mean hemifacial spasm duration**	**0–4 weeks**	**4–8 weeks**	**8–12 weeks**	**0–12 weeks**
**Neu-Botulinum toxin A (hours per day)**				
Mean ± Standard Deviation	2.83 ± 0.13	2.68 ± 0.04	3.36 ± 0.09	2.96 ± 0.06
Median [Interquartile Range: IQR]	2.65 [0.65]	2.61 [0.37]	3.12 [0.86]	2.92 [0.55]
Quartile 1–Quartile 3	2.33–2.98	2.54–2.91	2.93–3.79	2.59–3.15
**Abo-Botulinum toxin A (hours per day)**				
Mean ± Standard Deviation	4.73 ± 0.16	5.03 ± 0.09	6.06 ± 0.13	5.27 ± 0.10
Median [Interquartile Range: IQR]	4.63 [1.48]	4.83 [0.51]	5.74 [0.58]	5.67 [1.02]
Quartile 1–Quartile 3	3.92–5.41	4.79–5.30	5.68–6.27	4.83–5.86
***p*** **value**	**<0.001**	**<0.001**	**<0.001**	**<0.001**
**Absolute disability duration with hemifacial spasm**	**0–4 weeks**	**4–8 weeks**	**8–12 weeks**	**0–12 weeks**
**Neu-Botulinum toxin A (hours per day)**				
Mean ± Standard Deviation	0.01 ± 0.00	0.16 ± 0.03	0.42 ± 0.02	0.19 ± 0.02
Median [Interquartile Range: IQR]	0.00 [0.00]	0.17 [0.33]	0.34 [0.23]	0.17 [0.33]
Quartile 1–Quartile 3	0.00–0.02	0.00–0.33	0.33–0.57	0.00–0.33
**Abo-Botulinum toxin A (hours per day)**				
Mean ± Standard Deviation	0.00 ± 0.00	0.05 ± 0.01	0.03 ± 0.00	0.02 ± 0.00
Median [Interquartile Range: IQR]	0.00 [0.00]	0.00 [0.00]	0.03 [0.00]	0.00 [0.03]
Quartile 1–Quartile 3	0.00–0.00	0.00–0.18	0.03–0.03	0.00–0.03
***p*** **value**	**<0.001**	**<0.001**	**<0.001**	**<0.001**
**Improved sleep duration**	**0–4 weeks**	**4–8 weeks**	**8–12 weeks**	**0–12 weeks**
**Neu-Botulinum toxin A (hours per day)**				
Mean–Standard Deviation	1.29 ± 0.04	1.41 ± 0.00	1.41 ± 0.00	1.37 ± 0.01
Median [Interquartile Range: IQR]	1.34 [0.02]	1.41 [0.07]	1.41 [0.00]	1.41 [0.07]
Quartile 1–Quartile 3	1.32–1.34	1.36–1.44	1.41–1.41	1.34–1.41
**Abo-Botulinum toxin A (hours per day)**				
Mean ± Standard Deviation	1.07 ± 0.04	1.05 ± 0.00	1.05 ± 0.00	1.06 ± 0.01
Median [Interquartile Range: IQR]	1.05 [0.07]	1.03 [0.05]	1.05 [0.00]	1.05 [0.03]
Quartile 1–Quartile 3	1.05–1.13	1.03–1.08	1.05–1.05	1.04–1.07
***p*** **value**	**<0.001**	**<0.001**	**<0.001**	**<0.001**

**Figure A1:**
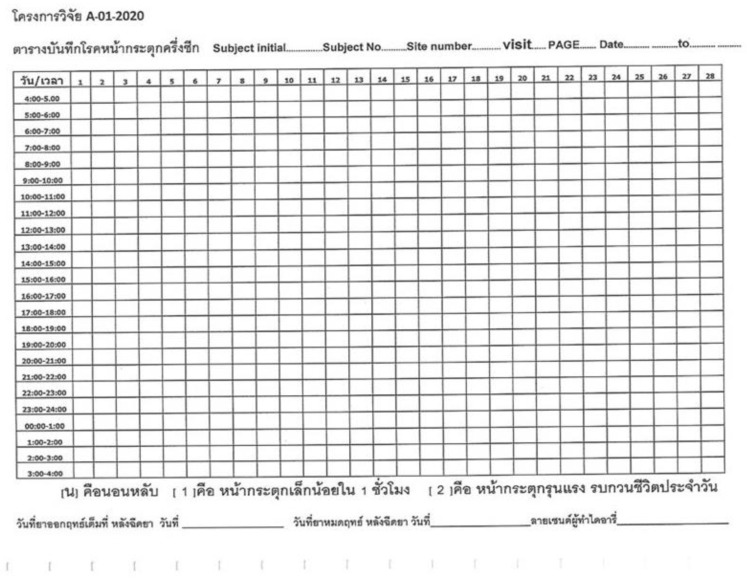
Patient Diary Description.

## Data Availability

The original contributions presented in this study are included in the article. Further inquiries can be directed to the corresponding author.

## Appendix A

The patient diary for study protocol A-01-2020 was designed to record hemifacial spasm symptoms in the Thai language. The diary table includes hourly entries from 4:00–5:00 a.m. to 3:00–4:00 a.m. the following day, with a total of 28 days recorded per diary. The Thai letter “น” is used to denote sleep time in each corresponding cell. The number “1” represents hemifacial spasm without disability. The number “2” represents hemifacial spasm with disability. Periods without hemifacial spasm symptoms are left blank in the table. The date of treatment onset and the date marking the end of the treatment effect are recorded on the bottom line of the diary. Patients personally sign the completed diary to confirm accuracy.
